# Absence of Cue-Recruitment for Extrinsic Signals: Sounds, Spots, and Swirling Dots Fail to Influence Perceived 3D Rotation Direction after Training

**DOI:** 10.1371/journal.pone.0013295

**Published:** 2010-10-08

**Authors:** Anshul Jain, Stuart Fuller, Benjamin T. Backus

**Affiliations:** Graduate Center for Vision Research, State University of New York College of Optometry, New York, New York, United States of America; University of Regensburg, Germany

## Abstract

The visual system can learn to use information in new ways to construct appearance. Thus, signals such as the location or translation direction of an ambiguously rotating wire frame cube, which are normally uninformative, can be learned as cues to determine the rotation direction [1]. This perceptual learning occurs when the formerly uninformative signal is statistically associated with long-trusted visual cues (such as binocular disparity) that disambiguate appearance during training. In previous demonstrations, the newly learned cue was *intrinsic* to the perceived object, in that the signal was conveyed by the same image elements as the object itself. Here we used *extrinsic* new signals and observed no learning. We correlated three new signals with long-trusted cues in the rotating cube paradigm: one crossmodal (an auditory signal) and two within modality (visual). Cue recruitment did not occur in any of these conditions, either in single sessions or in ten sessions across as many days. These results suggest that the intrinsic/extrinsic distinction is important for the perceptual system in determining whether it can learn and use new information from the environment to construct appearance. Extrinsic cues do have perceptual effects (e.g. the “bounce-pass” illusion [2] and McGurk effect [3]), so we speculate that extrinsic signals must be recruited for perception, but only if certain conditions are met. These conditions might specify the age of the observer, the strength of the long-trusted cues, or the amount of exposure to the correlation.

## Introduction

In order to construct useful representations of the environment from sensory data, the human visual system adapts itself to the environment in various ways. One such form of adaptation is the learning of perceptual biases, because sensory input to the perceptual system is not always sufficient to form a unique percept. Many 3-D scene structures can account for a given 2-D image on the retina. In the field of Perception, a “*cue*” is a *contingent bias*: it is a signal that can be measured in the sensory data that is informative about the state of the world, and can therefore be used by the perceptual system to select between alternative interpretations of the sensory data. For example, disparity between the retinal images in the two eyes and relative luminance differences across retinal locations are cues that can be (and are) used to construct apparent depth [4]. Some cues may have been recruited during evolutionary history via natural selection, so that one is predisposed to use them appropriately [5], while others are presumably learned during one's lifetime.

The adult visual system does in fact detect novel correlations in the environment and use them during perception [6], [7], [8]. This is exemplified by the phenomenon of “cue recruitment,” a form of classical conditioning [9]. In cue recruitment, the unconditional stimulus (US) is the long-trusted cue that is already effective to control some attribute of appearance (e.g., binocular disparity as a cue for depth). The conditioned or conditional stimulus (CS) is the “new signal” that may or may not come to be effective for controlling perceptual appearance (which is the conditioned response, or CR, in this case is the same as the UR).

Two difficulties in applying classical conditioning to the learning of a subjective mental state, such as visual appearance, are that we have neither the theory to link visual appearance to specific patterns of neural activity [10], nor the ability to measure such patterns of activity with precision. However, one can proceed with confidence if experimental conditions can be found in which subjects are easily able to follow the experimenter's instructions to report what they see. For that reason, we used short-duration stimuli that are perceptually bi-stable at onset and do not alternate within a given presentation [1], [11], [12], [13]. These stimuli force a dichotomous *perceptual* decision, so that observer responses are straightforward. Theory to quantify the effect of a cue on the appearance of such a stimulus, including the effect of a newly recruited cue, has been described elsewhere [11].

Haijiang et al. [1] used the cue recruitment paradigm to demonstrate that new signals can be adopted as cues to affect the appearance of perceptually bi-stable rotating wireframe (Necker) cubes. Such stimuli are useful in cue recruitment experiments because stereo-disparity can be used reliably to control their 3D appearance [1], [4], [12], [13]. Importantly, the removal of disparity and other cues renders the cubes perceptually ambiguous, and therefore well suited as probe stimuli to measure whether learning has occurred. After the initial conditioning or “training” period, the perceived rotation direction of the cube came to be influenced, in separate experiments, by either of two new signals: the cube's spatial position or its translation direction. In other words, spatial position and translation direction were new signals that were recruited to act as cues for 3D rotation by the observers' visual systems.

Interestingly, an auditory new signal was not recruited to function as a cue for appearance [1]. A restrictive interpretation of this finding would be that it stemmed from the cross-modal nature of the sensory relationship between the new signals and the cubes. However, there are well known examples of cross-modal interactions that alter the percepts in one or both modalities, such as the “bounce-pass” effect [2], the McGurk effect [3], the double-flash illusion [14], and the ventriloquism effect [15]. Moreover, Ernst [16] showed that the perceptual system can learn to integrate haptic and visual signals in a cue recruitment paradigm similar to that used by Haijiang [1]. In that study, observers performed a three-interval odd-man-out task on virtual objects that could differ in stiffness (haptic) or in brightness (visual) or both. During training phase, the stiffness and brightness of the object were perfectly correlated (stiffer objects were always brighter, or vice versa). After training, discrimination thresholds for objects with stiffness and brightness values incongruent with the training correlation were larger than for congruent objects. Taken together, these facts suggested to us that cross-modality per se did not impede learning in Haijiang et al. [1].

Another interesting distinction between the recruited and unrecruited signals in Haijiang et al [1] is that the former were *intrinsic* to the cube itself, whereas the latter were not. Specifically, the recruited cues were conveyed by the same image elements as the object whose perceptual appearance was affected. The current study was designed to determine whether new *extrinsic* signals (i.e. signals that are not carried by the object itself) could be learned for perceiving properties of the object. Specifically, we tested whether physically plausible auditory signals (Experiment 1), and two additional types of extrinsic visual signals (location of a luminous disc and rotation direction of a random-dot field, Experiments 2A and 2B), could be learned and used to disambiguate the rotation direction of an ambiguous cube.

The experiments can be seen within a general framework of classical conditioning, wherein one can choose an arbitrary new signal and try to train it, by means of association, to become effective at controlling some arbitrary attribute of appearance. The learning may or may not then occur, i.e., the signal may or may not actually be recruited so that it comes to control the attribute of appearance. One can then look for patterns: what sorts of pairs can be learned, and what sorts cannot [17], [18]? Here we asked: Does it matter if the new signal is from the same sensory modality as the long-trusted cues? If both are visual, does it matter whether the new signal is intrinsic to the object? These questions are interesting because it would be inefficient, in principle, for the system to learn any arbitrary association. The capability to do so requires monitoring correlations for an extraordinarily large set of signal/attribute pairs, and some associations are so unlikely to be causal that the system would be well served to treat them as accidental. In the real world, for example, we might presume that the system would be ill advised to use big toe temperature as a cue for apparent depth between two objects; any correlation is presumably due to chance.

**Figure 1:**
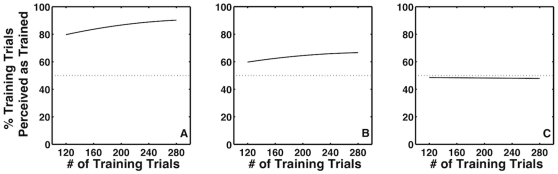
Expected form of the data if the new signals are recruited strongly (A), weakly (B) or not at all (C). The figures were generated based on the size of the effect observed in [1].

**Figure 2:**
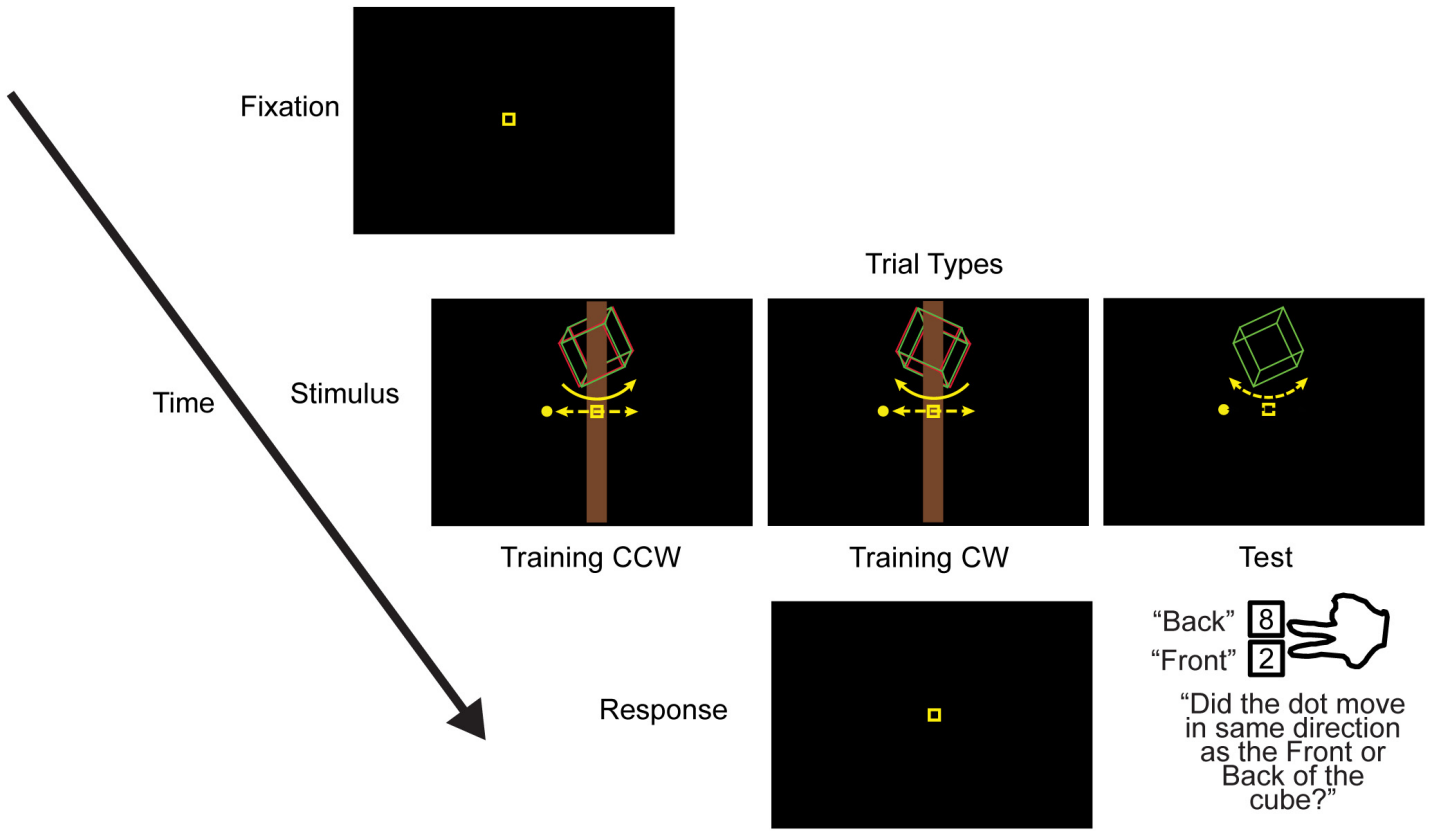
The structure of a typical trial presented during the experiments.

**Figure 3:**
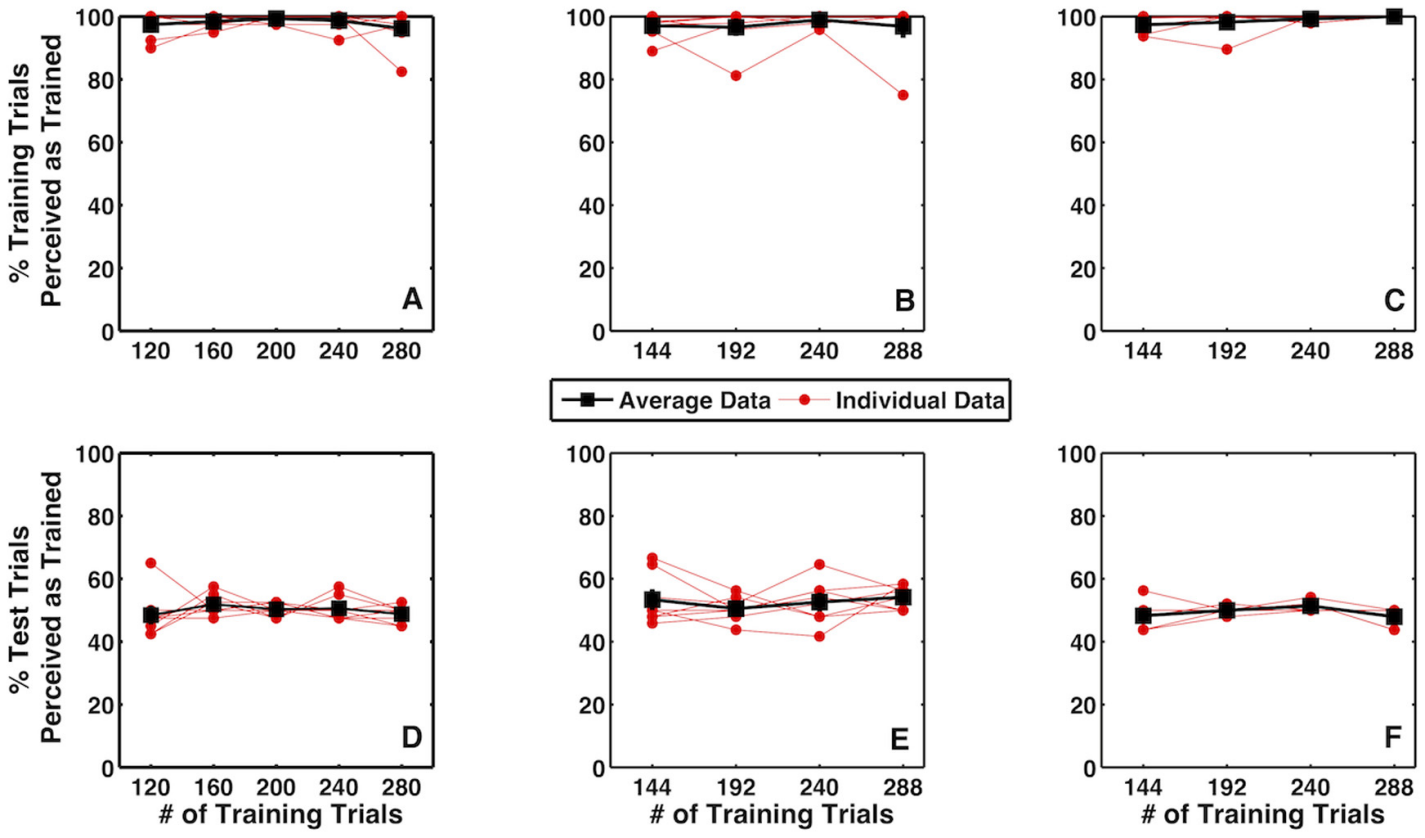
Percent accuracy on training trials during training for auditory (A), stationary disc (B) and rotating random-dot field (C) training signals. Percent of test trials perceived as predicted by training as a function of training trials for auditory (D), stationary disc (E) and rotating random-dot field (F) training signals.

Thus, in the current study, the associative training could lead to one of three possible outcomes, independently for each of the three new signals: 1) the new signal is recruited strongly (like the position signal in [1], depicted in Figure 1A), or 2) the new signal is recruited weakly (like the translation signal in [1], depicted in Figure 1B), or 3) the new signal is not recruited at all (like the auditory signal in [1], depicted in Figure 1C). In the first two cases, the new signal would come to affect the apparent rotation direction of the ambiguous cubes after training while in the last case the new signal would have no effect on the apparent rotation direction of the cube. As it happened, none of our three extrinsic signals were recruited. There are several possible reasons for the lack of recruitment and we consider them in the Discussion.

## Materials and Methods

### Subjects

Twenty-two subjects participated in the experiments, eight in Experiment 1, eight in Experiment 2a, and six in Experiment 2b. All subjects were naïve to the purpose of the experiments. The experiments were conducted in compliance with the standards set by the IRB at SUNY State College of Optometry. Subjects gave their written informed consent prior to their inclusion in the study and were paid for their participation. All experimental procedures were approved by the IRB. All had normal or corrected-to-normal vision and normal hearing (self reported). We assessed each subject's stereo-acuity using the TNO Stereo-acuity test to validate their ability to see the stereo-disambiguated training cubes as we intended; all subjects had a stereo-acuity of at least 120 seconds of arc.

### Apparatus

The experiments were implemented on a Dell Precision 3400 computer (Windows platform) using the Python-based virtual reality toolkit Vizard™ 3.0 (WorldViz LLC, Santa Barbara, CA, USA). Visual stimuli were back-projected onto a screen using either a Cristie Mirage S+ 4 K projector (auditory cue recruitment experiments, Experiment 1) or an Infocus LP350 projector (visual cue recruitment experiments, Experiment 2). Auditory stimuli were presented on Bose® 161™ speaker system that were driven by AudioSource Stereo Amplifier AMP-100. The speakers were placed on either side of the screen along the horizontal midline. Subjects were seated at a distance of 2 m from the screen for Experiment 1 and at a distance of 1.3 m from the screen for Experiment 2. The experiments were conducted in separate rooms. In Experiment 2 we used an EyeLink I™ eye tracker (Missisauga, Ontario, CA.) to monitor fixation and record eye position. Given that previous studies showed a strong retinotopic constraint on learning [12], [13], we recorded gaze coordinates and provided observers feedback on their eye fixation, to avoid potentially diluting any learning due to eye movements and inconsistent retinal positioning of the stimuli (see Display).

### Display and Stimuli

#### Visual Task Stimulus

The visual stimulus consisted of an orthographically projected (i.e. no perspective) wireframe (Necker) cube rotating about the vertical axis. The edges of the cube were 25 cm long for Experiment 1 and 15 cm long for Experiment 2. Edges were rectangular parallelepipeds with a thickness of 0.6 cm for Experiment 1 and 0.3 cm for Experiment 2. The cube subtended 12.4 degrees of visual angle. In order to stabilize perception of the cube as a single rigid object, each face of the cube was covered with 25 randomly placed dots. The cube was oriented such that one of the major diagonals was perpendicular to the axis of rotation. The cube was presented in two configurations, as “seen-from-above” or as “seen-from-below”. The yaw, pitch and roll were set to 50, 25 and 25 degrees respectively at the onset for “seen-from-above” configuration and at 50, −25 and −25 degrees for “seen-from-below” configuration. These two configurations were balanced across both test and training trials to avoid correlating them with cube rotation. The cube was rotated about the vertical axis at 60 degrees/second during Experiment 1 and 72 degrees/second during Experiment 2. On training trials, the rotation direction was disambiguated using disparity and occlusion. The disparity was calculated for 6.2 cm inter-pupillary distance and fusion at the plane of the screen and was implemented using red-green anaglyphs (observers wore red/green filter glasses during the experiments). The occluder was an opaque vertical column that passed through the center of the cube, spanning the entire height of the screen and with a diameter of 7 cm for Experiment 1 and 4 cm for Experiment 2. On test trials only one of the anaglyph images of the cube was presented (to the right eye), and the occluder column was omitted. During the experiment, there was a fixation square (2 cm×2 cm) presented at the center of the screen. The cube was centered 25 cm (7.12 degrees) above the fixation square during Experiment 1 and 15 cm (7.12 degrees) above the fixation square during Experiment 2. The cube's center was simulated to be in the plane of the screen. All stimuli were presented as bright objects on a dark background.

Concurrent with the cube stimulus on each trial, we presented a single probe dot (1 cm×1 cm) that translated horizontally in the screen plane through the fixation point, either leftwards or rightwards across a visual angle slightly larger than the cube. The dot traveled at approximately the same speed as the closest (or farthest) corner of the cube.

#### “New Signals”

We use the term “new signals” to designate the visual and audio elements in the various experiments that are correlated during training with the rotation direction of the disambiguated stereo cube stimulus, i.e. the candidates for recruitment as cues. As described previously, the goal of the experiments is to investigate whether the visual system can learn to use the presence of these “new signals” to resolve ambiguous 2D test cubes based on the correlations during training.

In Experiment 1, we used two auditory new signals, which were paired with the two directions of cube rotation during training (counterbalanced order across subjects). We hypothesized from Haijiang et al. [1] that recruitment of auditory cues for visual appearance might require some physical or cognitive correspondence between the sound and the visual object. We selected a “ratchet” sound (Audio S1) and a “camera-film winding” sound (Audio S2) as the auditory signals because they are easily distinguishable from each other and associated with rotating objects in physical world. The sound intensity in the two speakers was balanced and the speakers were placed along the horizontal midline of the screen, in order to evoke a perceived location of the sound source at the visual location of the cube and encourage physical correspondence. Indeed, motion detection thresholds for audiovisual stimuli are lowest when the auditory and visual components are concurrent and co-localized, presumably because such cross-modal stimuli are treated as emanating from the same object [19]. Finally, we attempted to further increase correspondence between the cube and the sounds by “stuttering” their presentations in tandem (15 epochs of 50 ms motion separated by progressively shorter stationary intervals of 150-10 ms, after which the motion was continuous; see Movie S1). The sounds were downloaded from a free online sound database (www.sounddogs.com, monaural, sampling rate 22.05 kHz, bit-rate 352 kbps) and were edited using GoldWave (GoldWave Inc., Canada) audio editing software to exclude any blank periods and large intensity modulations. The intensity was supra-threshold for both auditory stimuli (the exact intensity is not critical to the aim of the study).

In Experiment 2a, the new signal was a stationary disc located to either side of the rotating cube. The correlation of disc location (right or left) and rotation direction was counter-balanced across subjects. The disc was located at the same height as the cube and with a horizontal offset of 30 cm (14.25 degrees). The disc was 13.5 cm (6.42 degrees) in diameter. The minimum distance between the cube and the disc was approximately five degrees of visual angle. The stimuli were presented only after verifying subjects' fixation via the eye-tracker. Once the subject fixated for 500 ms, the disc was presented on the right or left of the fixation square followed by the rotating cube with a stimulus onset asynchrony of 50 ms (see Movie S2).

In Experiment 2b, the new signal was an annulus of randomly placed dots surrounding the cube. The annulus rotated at the same angular speed as the cube and the rotation direction was perfectly correlated with the cube's rotation direction on training trials. The polarity of the correlation was counterbalanced across subjects. The dots had a mean lifetime of 100 ms. The field rotated at the same angular speed as the cube. The field of dots and the cube were presented simultaneously after ascertaining fixation (see Movie S3).

### Procedure

#### General Procedure

The experiments consisted of two kinds of trials, Training trials and Test trials. On training trials, the perceived rotation direction of the cube was controlled using stereo and occlusion cues as described above (see Movie S1) to establish the correlation with the new signal. On test trials the cube was ambiguous (no disparity or occluder) and presented with the same new signal. If the new signal were adopted by the perceptual system to aid in visual resolution of the ambiguous test cubes, then over time the test cube's rotation direction should become correlated with the new signal, with the same contingency as in the training trials (Figure 1). Subjects were instructed to fixate on the fixation square throughout the experiment. We monitored fixation during Experiment 2 (visual new signals) but not in Experiment 1 (auditory new signal) since the display did not include visual elements other than those used for the task that might attract gaze. The eye-tracker was recalibrated before each of the five trial blocks in Experiment 2.

A trial consisted of the presentation of the cube stimulus, the probe dot, and the new signal corresponding to the particular experiment (2 s Experiment 1, 2.5 s Experiment 2). The subjects' task was to report whether the translation direction of the probe dot was same as the front (closer to the subject) or back (farther away from the subject) side of the cube. Because the dot's direction was randomly chosen on each trial, subject responses were decoupled from both perceived rotation direction and the new signals' values (e.g. disc location was on the right or left of the cube). At the end of a trial, we presented a text message at the center of the screen reminding them of the meaning of the response keys: the ‘2’ key to report that the front of the cube moved in the same direction as the probe dot or the ‘8’ key to report that the back of the cube moved in the same direction as the probe dot (Figure 2). The task was chosen to discourage subjects from “figuring out” the experiment or using complicated cognitive strategies. Post-experiment interviews confirmed that responses were mediated by the apparent rotation of the cube: none of the 22 subjects reported having noticed the correlation of training signal and rotation direction. The next trial began 1 s after response.

A single session of the experiment consisted of 480 trials split into six blocks of 80 trials each for Experiment 1 and five blocks of 96 trials each for Experiment 2. The first block contained only training trials, to establish a perceptual history reflecting the correlation of the new signal and cube rotation before beginning to test with ambiguous cubes. The training trials for rightward and leftward rotations were presented equally often but pseudo-randomly sequenced. The sequence was constrained such that subjects could not be presented with cubes rotating in the same direction on more than eight consecutive trials. The remaining blocks contained an equal mix of test and training trials presented pseudo-randomly. The sequence was constrained such that subjects could never be presented with the same type of trial on more than four consecutive trials.

#### Ten-day Procedure

Anticipating the possibility that more training time and effort may be required to manifest learning than in previous experiments [1], [12], [13] we ran three subjects in modified ten-day versions of Experiments 1 and 2. Here we describe only the modifications, all other aspects were the same as described above. First, subjects ran one-hour sessions identically structured to those described above, each weekday for two weeks. One subject saw the dot-annulus, and two were presented with auditory new signals. Second, we slightly modified the auditory new signals for the ten-day subjects to incorporate a directional sweep between the two audio speakers on either side of the screen (e.g. ratchet + leftward sweep/film wind + rightward sweep, or vice versa). The motivation for this change was speculation that having spatial motion present in both the audio signal and the rotating training cubes might facilitate learning of the correlation between the two. The auditory sweep was achieved by progressively increasing the sound intensity in one speaker while simultaneously decreasing the sound intensity in the other.

## Results and Discussion

For statistical analyses, each observer's percent accuracy on test and training trials was converted to a z-score measure [4], [11]. The performance on ambiguous test trials was computed based on the expected response as predicted by the new signal contingency during training. Saturated performances (0% and 100%) were assigned a z-score of ±2.326. This z-score corresponds to 2 incorrect/correct responses in 200 trials. The confidence intervals (95%) were computed using bootstrapping method.

### Absence of learning

The stereo and occlusion cues were effective in controlling subjects' percept of the cubes on the training trials. Subjects performed with a mean accuracy of 98% (z-score  = 2.07, 95% CI [2.05 2.30], t(7) = 16.67, p<0.0001) on the training trials in Experiment 1, 96.7% (z-score  = 2.02, 95% CI [1.94 2.15], t(7) = 13.23, p<0.0001) on the training trials in Experiment 2a and 98.5% (z-score  = 2.18, 95% CI [2.05 2.27], t(5) = 14.58, p<0.0001) on the training trials in Experiment 2b. Subjects maintained fixation well during Experiment 2a and 2b, with breaks in fixation occurring on fewer than 1% of the trials on average for all subjects (maximum across subjects 1.7%).

Figure 3 shows the percentage of training (panels A–C) and test trials (panels D–F) that were judged as rotating in the direction predicted by training as a function of the number of training trials for the three experiments. All subjects responded at close to chance levels (50%) on the test trials, showing that there was no cue recruitment for any of the three new signals. The mean proportions of test trial responses that were consistent with the contingency between new signals and training cube rotations were ∼50% (z-score ∼ = 0, 95% CI [−0.06, 0.06], t(7) = 0, p>0.99) in Experiment 1, 52.7% (z-score  = 0.07, 95% CI [0.00, 0.13], t(7) = 2.15, p = 0.07) in Experiment 2a and 49.4% (z-score  = −0.02, 95% CI [−0.09, 0.06], t(5) = −0.78, p = 0.47) in Experiment 2b.

**Figure 4:**
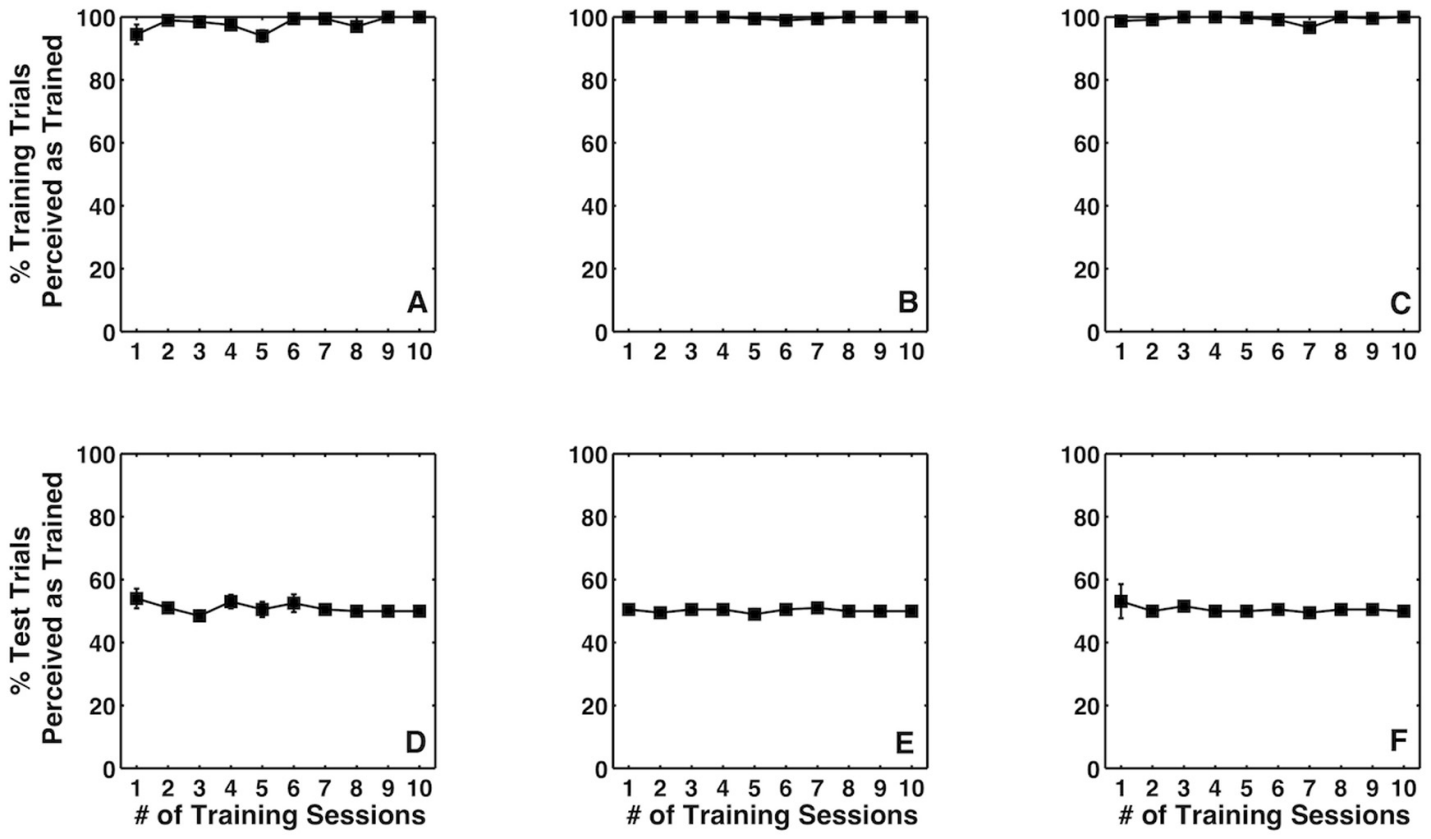
Percent accuracy on training trials for auditory signals (two observers in panel A and B respectively) and rotating random-dot field (single observer in panel C) training signals. Percent of test trials perceived as predicted by training as a function of training sessions for auditory signals (two observers in panel D and E respectively) and rotating random-dot field (single observer in panel F) training signals. Data were averaged across training blocks.

Further, we did not observe any learning even when observers underwent training for ten sessions. Observers' performance on training trials was extremely consistent throughout the training. The mean training accuracy for the two observers that participated in auditory training was 98.7% (z-score  = 2.22, 95% CI [2.1 2.29]) and 99.1% (z-score  = 2.37, 95% CI [2.30 2.45]), respectively. The mean training accuracy for the observer who participated in the 10-day training with random-dot field as the new signal was 99.1% (z-score  = 2.36, 95% CI [2.24 2.41]). However, the performance on test trials was 51% (z-score  = 0.023, 95% CI [−0.03 0.08]) and 50.9% (z-score  = 0.02, 95% CI [−0.05 0.06]) for the two observers that participated in auditory training and 50.6% (z-score  = 0.01, 95% CI [−0.04 0.07]) for the observer who participated in random-dot-field training. Figure 4 shows the performances of the three observers on training trials (panels A–C) and test trials (panels D–F).

### Was learning masked by stabilization?

Repeated intermittent presentation of a rotating cube, at a given location in the visual field, leads to both short term priming [20], [21], [22], [23], [24] and to long term learning of a bias [25] at that location. In our experiments here, most observers saw the cube rotate in the same direction (either leftward or rightward) on almost all test trials, resulting in a performance that was close to 50% on test trials. In other words, the new signal did not influence the perceived rotation direction and the observers tended to follow the percept as determined by short-term priming and their internal bias. Training stimuli were shown rotating in both directions, but a bias to perceive test trials rotating one way or the other might accumulate over time, with test trials influencing future test trials to be seen the same way [25]. This raises the question: was learning of the new cues masked by a ceiling effect due to strong overall bias? This seems unlikely, because some subjects did show biases that varied over time, and in these cases the bias co-varied for the two cues across the session (see Figure S1). These subjects strongly support our basic claim that the new signals were not learned. In any case, it is clear that unlike previously tested intrinsic signals, extrinsic signals were not learned to the point of having measurable effects using the current paradigm.

### Why not extrinsic cues?

The visual system acted as though extrinsic signals were unlikely to be valid cues for object properties, even though these signals perfectly predicted the object property, and hence no learning occurred. Our expectation, that extrinsic new signals would be recruited to act as cues, was supported by the existence of cross-modal and unimodal interactions between perceptual signals. Indeed, we took steps to encourage perceptual congruence between the audio signals and visual stimuli in Experiment 1. Despite the fact that the visual new signals used in Experiments 2a and 2b were separate objects from the cubes, spatial and temporal integration in the visual system are well known, including the use of information from spatially separate locations to resolve ambiguities (e.g.[26], [27], [28], [29], [30]). Regarding our rotating annulus, there are many examples of perceptual linkages between the motions of multiple objects when at least one is ambiguous [31], [32], [33], [34], [35]. We had also reasoned that the global rotation of the annulus was of the sort that would excite neurons in the cortical area hMST [36], [37]. Such neurons are known to have large receptive fields [38], potentially encompassing both the annulus and cube and thus possibly serving as a neural locus for recognizing and learning the statistical association between the new signals and cube rotation [39].

It is possible that the determining factors here could have been experimental, e.g. learning may have occurred at a rate too slow for us to measure, or only with a great deal of exposure that is difficult to simulate in the laboratory. In the case of perceptual learning that causes improved discrimination between similar stimuli, learning sometimes occurs very quickly, after several trials [40], and sometimes more slowly, over several hours [41] or several days [42]. Fine and Jacobs [43] have identified stimulus and task properties that are associated with various rates of learning in discrimination tasks. Thus, it would not be surprising to find different rates of learning as a function of what is being learned in a cue recruitment task, including perhaps rates of learning that are too slow to be measured within a few sessions.

Another way to approach the question is to ask what is different about perceptual interactions such as bounce-pass and McGurk effects, specifically relative to our sound signals. One possibility is that these particular auditory/visual correlations are frequently encountered in the natural environment, starting from a young age, and/or that our perceptual systems are predisposed to accept them (perhaps *because* they are common). Speech interpretation is a very important, yet complex task, for which it is useful to incorporate additional reliable visual information that is attributable to the same source. By implication, our assignment of sounds to cube rotations must not have been deemed by the system to be sufficiently important or reliable to be learned. For our visual cues, we speculate that the failure of cue recruitment was a casualty of the way visual system deals with the conflicting challenges of grouping elements into objects (assimilation) and segregating objects from one another (differentiation). The visual cues we offered were unquestionably separate objects. Our results suggest that statistical congruence between separate objects, no matter how reliable, is not a sufficient condition to modify the system's apparent default that individual objects in the environment are independent.

## Supporting Information

Movie S1 The movie shows stimuli from Experiment 1 (Auditory Cue). A Clockwise Training trial, Counter-clockwise Training trial and a Test trial are depicted sequentially.
(3.00 MB MOV)

Movie S2 The movie shows stimuli from Experiment 2a (Stationary Disc Cue). A Clockwise Training trial, Counter-clockwise Training trial and a Test trial are depicted sequentially.
(0.67 MB MOV)

Movie S3 The movie shows stimuli from Experiment 2b (Swirling Dots Cue). A Clockwise Training trial, Counter-clockwise Training trial and a Test trial are depicted sequentially.
(2.06 MB MOV)

Figure S1 Percentage of test trials perceived as rotating clockwise as a function of training trials for clockwise (black squares) and counter-clockwise (red circles) for eight observers in Experiment 1. As can be seen in the figure, some subjects (S1, S5, S8) showed biases that varied significantly across the session, but this variation occurred in tandem for the two cues.
(6.48 MB TIF)

Audio S1 This is the audio of the "Ratchet" auditory signal used in Experiment 1.
(0.73 MB WAV)

Audio S2 This is the audio of the "Camera-film winding" auditory signal used in Experiment 1.
(0.37 MB WAV)
