# Supplementary figures and images for: Absence of Cue-Recruitment for Extrinsic Signals: Sounds, Spots, and Swirling Dots Fail to Influence Perceived 3D Rotation Direction after Training
Source: PLoS One. 2010 Oct 8;5(10):e13295. doi: 10.1371/journal.pone.0013295 (PMC2951915; doi:10.1371/journal.pone.0013295)

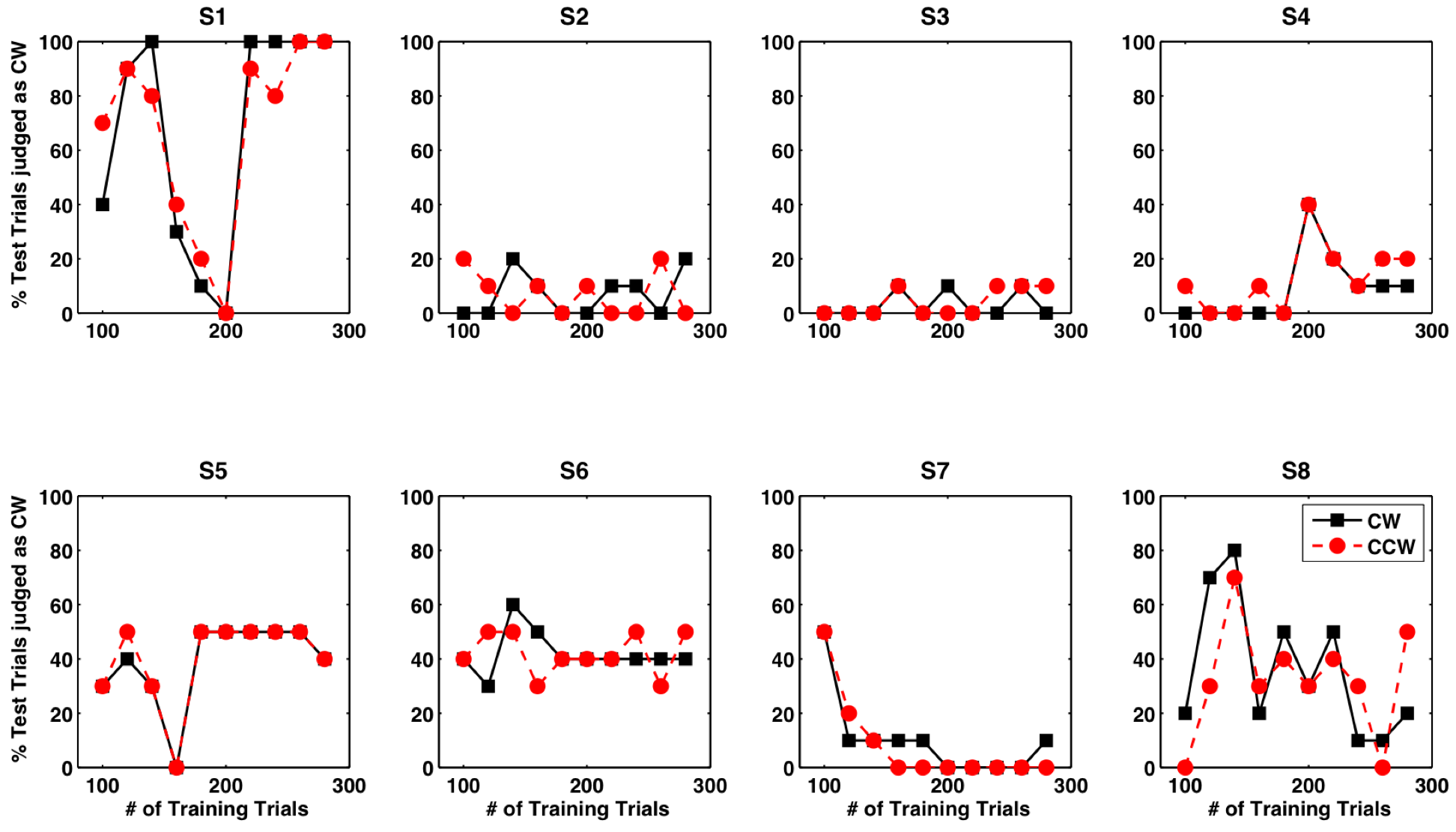

Supplement: Figure S1 — Percentage of test trials perceived as rotating clockwise as a function of training trials for clockwise (black squares) and counter-clockwise (red circles) for eight observers in Experiment 1. As can be seen in the figure, some subjects (S1, S5, S8) showed biases that varied significantly across the session, but this variation occurred in tandem for the two cues. (6.48 MB TIF) [file pone.0013295.s004.tif]
